# Supplementary material for: Endoparasitism of Golden Retrievers: Prevalence, risk factors, and associated clinicopathologic changes
Source: PLoS One. 2022 Feb 18;17(2):e0263517. doi: 10.1371/journal.pone.0263517 (PMC8856576; doi:10.1371/journal.pone.0263517)
Supplement: Attachment — Submitted filename: Dear member of PLOS.docx [file pone.0263517.s003.docx]

Sun Aug 1, 2021

**Dear member of PLOS**

Here you will find some of the recommendations for the article titled “Intestinal parasitism of Golden Retrievers: prevalence, risk factors, and associated clinicopathologic changes” I recommend publishing, improving the quality of the manuscript and discussion.

The study has important, relevant data that help explore future methods or alternatives to assess the impact of low parasite load (<4 eggs per gram) on feces, unfortunately, it is not well written, and the relevant data is not well discussed. I recommend supporting data with studies done to understand subclinical parasite effects with other models and populations: <https://doi.org/10.3389/fimmu.2018.02975>. Systemic Cytokine and Chemokine Profiles in Individuals With Schistosoma mansoni Infection and Low Parasite Burden.

**Tittle**

The title needs to be consistent in all the manuscripts: intestinal, gastrointestinal, or endoparasites. I will recommend changing to “endoparasites.”

**Abstract**

The study titled “Intestinal parasitism of Golden Retrievers: prevalence, risk factors, and associated clinicopathologic changes” is a retrospective study with a representative data analysis that could be used to compare with other dog populations in rural and urban areas. However, the assumption that “dogs living in rural areas were more likely to have endoparasites than those living in suburban areas” needs to be removed, considering that the study was not focused on this issue and there is not enough knowledge to suggest this in the abstract.

**Abstract.**

Line 33-37 I recommend “the identification of endoparasites by fecal testing is an essential tool to identify susceptible and resistant animals that can act as spreaders and reservoirs of intestinal parasites thereby enabling appropriate therapy and reducing the risk of new infection to animals and humans. It is crucial information for further epidemiological studies and to prevent and monitor and develop new strategies to control.

**Author summary**

Line 47-49. “Our data suggest that subtle changes may be seen on routine blood work submitted on young dogs that should prompt further investigation to diagnose or rule out endoparasitism” I recommend adding “and develop coprological serological or molecular tests to get stronger epidemiological information.”

**Introduction**

Line 62-63. The same for the title here, I recommend Endoparasites rather than Gastrointestinal.

Line 63-66. As the work uses coprological methods, I recommend citing references that have been used. These methods described are helpful in “availability of inexpensive diagnostic tests and effective treatments” for epidemiological records in developed and undeveloped countries such as “Peña Quistial et al., 2020. **Prevalence and associated risk factors of Intestinal parasites in rural high-mountain communities of the Valle del Cauca—Colombia**

Line 63-66. **I recommend highlighting** how using complementary techniques can be helpful too. However, it is not necessary to arrive at a post mortem examination to get a better diagnosis as given the idea following ….” Prevalence of infection with at least one intestinal parasite has been reported in up to 77.3% of dogs on post-mortem examination in a 2017 study, many of which had no gastrointestinal signs at the time of death[1]”.

Line 66-67. In this phrase, authors need to be careful “The continuous fecal shedding of infective ova from undiagnosed or untreated dogs allows for the propagation of parasitic infection to other susceptible animals.” There are two kinds of dog populations: susceptible and resistant populations; all of them spread parasites. The difference is that resistance is asymptomatic and silently keeps parasites while susceptible will develop symptoms, and both will spread parasites to other animals’ populations, both susceptible and resistant again. I suggest a reference that can support this concept much better.

Line 68-71. I recommend talking that there is an enormous number of zoonotic parasites, protozoa and helminth that give a better idea of the risk for humans and animals “Furthermore, the zoonotic risk of several parasites adds further importance to the prompt identification of parasitic infections. An important example of this is Ancylostoma spp. and Toxocara spp., which cause cutaneous larva migrans and visceral or ocular larval migrans respectively [2]”.

Line 71-72 The author arrives at One Health Approach suddenly without context as the solution to reduce asymptomatic infections, so this idea needs to be introduced in a better way “Reduction of asymptomatic infections on both companion animals, and human health can be achieved through a one health approach”.

Line 79-80 “animals residing across the United States was published in 2009 [8], but there have been no updated studies in the most recent decade”. This study updates the information in the USA after a decade, so I think that needs to be in the abstract.

Line 84-89. The way as has been written is not clear first studies support severe parasitism and in the absence of gastrointestinal signs…and after clinicopathological changes associated with low infection level in asymptomatic animals has not been documented (maybe could be improved, please clarify) “Several clinicopathological changes associated with severe parasitism have been documented and include anemia, hypoalbuminemia, and eosinophilia [9-12]. Previously published literature has also reported that endoparasitic infections can be present in the absence of gastrointestinal signs [2-5]. However, information regarding the impact on animal well-being is limited, and a comprehensive assessment of clinicopathological changes associated with infection levels in a large cohort has not been completed in the recent decade.

Line 90-95. Here the idea is that preventive medicine needs to be applied based on evidence, and parasite deworming needs to be improved, detecting asymptomatic animals, reducing zoonotic and animal transmission. (Please improve the writing) “Identifying risk factors and clinicopathological changes associated with endoparasite infections would provide an additional clinical tool for veterinarians in their assessment of dogs for additional fecal testing or empirical deworming. This has the opportunity to benefit both the patient and the larger canine population as the infection would be treated, preventing transmission to other dogs. For parasites of zoonotic importance, human transmission risk would also be reduced”.

Line 96 -101. Here in summary, the authors did a retrospective study or a cohort study associating clinical data with endoparasite infection, but how the study let to achieve this hypothesis is not clear as well as how the study tests the density of the populations as a risk factor to spread parasites is not clear? The objective of this study was to determine the prevalence of intestinal parasites within a large canine cohort and evaluate clinicopathologic data for any changes associated with endoparasite infections. We hypothesized that alterations in CBC and biochemical parameters exist due to the presence of a parasitic infection and its associated immune response. Additionally, we hypothesized that residing in more densely populated regions may increase the likelihood of endoparasites infection.

**Introduction**

Line 62-63. The same for the title here, I recommend Endoparasites rather than Gastrointestinal.

Line 63-66. As the work uses coprological methods, I recommend citing references that have been used. These methods described are helpful in “availability of inexpensive diagnostic tests and effective treatments” for epidemiological records in developed and undeveloped countries such as “Peña Quistial et al., 2020. **Prevalence and associated risk factors of Intestinal parasites in rural high-mountain communities of the Valle del Cauca—Colombia**

Line 63-66. **I recommend highlighting** how using complementary techniques can be helpful. However, it is not necessary to arrive at a post mortem examination to get a better diagnosis as given the idea following ….” Prevalence of infection with at least one intestinal parasite has been reported in up to 77.3% of dogs on post-mortem examination in a 2017 study, many of which had no gastrointestinal signs at the time of death[1]”.

**Materials and Methods**

Line 259 case selection. The way the cases were selected is clear and the information available.

Line 273 Laboratory.

Line 280 Statistical modeling is appropriately described

**Results.** Results are correctly described

**Discussion.**

Line 147-148 “Endoparasitism remains common in dogs in the United States despite frequent empiric treatment of puppies and routine fecal examinations”. So why is the treatment of puppies in the USA empiric, there is no diagnosis or treatment based on evidence? So it is possible to be more precise.

Line 153-155. Here, the authors need to clarify that it is not in Canada or Australia studies in this study. Is this correct?. Multiple statistically significant, albeit modest, clinicopathologic changes were identified between patients who were positive via fecal flotation for at least one pathogenic parasite when compared to patients with no evidence of endoparasitism.

Line 157-162. In the discussion, there is no emphasis in the results. It is not consistent whether the reduced prevalence is the effect of medical care and people conscious of zoonotic risk factors or the sum of all of them and the broad-spectrum parasite. After that, minimize the test saying that the data is not so true prevalence. I will say that the test has limited sensitivity but still is available to see the iceberg. So please be more consistent about what you want to say. “The reduced prevalence may indicate increasing awareness of endoparasitism and its zoonotic potential as well as the implementation of routine administration of broad-spectrum anthelmintics. Prophylactic anthelmintic and the use of preventative care are increased in the GRLS population [15]. Additionally, as with previous prevalence studies, the true prevalence of parasite infection is likely higher than reported values due to the limited sensitivity of fecal flotation [16]”.

Line 164-174 Some arguments in the document could be much better discussed and supported in the literature, such as the Review “The immune response to parasitic helminths of veterinary importance and its potential manipulation for future vaccine control strategies. [Neil Foster](https://link.springer.com/article/10.1007/s00436-012-2832-y#auth-Neil-Foster) & [Hany M. Elsheikha](https://link.springer.com/article/10.1007/s00436-012-2832-y#auth-Hany_M_-Elsheikha)” DOI 10.1007/s00436-012-2832-y

**Conclusions**

Are correctly address
