# Supplementary material for: Endoparasitism of Golden Retrievers: Prevalence, risk factors, and associated clinicopathologic changes
Source: PLoS One. 2022 Feb 18;17(2):e0263517. doi: 10.1371/journal.pone.0263517 (PMC8856576; doi:10.1371/journal.pone.0263517)
Supplement: Attachment — Submitted filename: Response to reviewers.docx [file pone.0263517.s005.docx]

Response to reviewers

- All data needs to be made available
  - Thank you for your comment – The data has been added as supplementary material to the manuscript and made available through 2 different online data sites (<https://data.world/ehales/grls-parasite-study> and https://datacommons.morrisanimalfoundation.org/).
- 1. The authors write that in future lab tests could be used to prioritize screening for GI parasites. However, I think they need to address the diagnostic value of the lab tests, and not just the statistical significance of the associations between lab tests and endoparasitism. It is clear from the data they provide that the magnitude of the effects are very small and there would be a large overlap in healthy and infected dogs. Also, they state a large proportion of cases may go undetected. Might it be more advisable to suggest screening and/or treatment of all young dogs?
  - Thank you for your comment, we agree and have changed the manuscript accordingly.
    - Line 186 we have added:
      - Additionally, given the small magnitude of difference identified between infected or not infected dogs and lack of specificity of these changes for endoparasitic infection, it is clear that identification of clinicopathologic differences does not replace regular screening fecal testing and empiric treatment of high-risk patients.
- 2. The authors infer that the parasites cause the lab test changes, but do not address alternative explanations that cannot be ruled out from this cross-sectional study design i.e. reverse causality or confounding. I think it would improve the manuscript if this was highlighted in the discussion.
  - Thank you for your comment, we agree and have changed the manuscript in several location including:
    - Line 56 reads:
      - Our data suggest that subtle changes may be seen on routine bloodwork submitted on young dogs and **those may be associated with a positive fecal float result.**
    - Line 200 reads:
      - Additionally, we cannot rule out the possibility that decreased albumin could have impacted the ability of the parasite to colonize an animal.
    - Line 255-258 now read:
      - Lastly, while every effort was made to eliminate dogs with concurrent disease processes, given the study design, we cannot prove the lab work changes identified are attributable entirely to endoparasitism. Additional follow up studies should be performed to confirm our findings and characterize the underlying molecular causes for the clinicopathologic changes seen in our study.
    - Lines 246-248 added:
      - An alternative, albeit considerably less likely explanation, would be that dogs with these preexisting clinicopathologic abnormalities have an increased susceptibility to endoparastitic infection.
    - Lines 180-183 added:
      - While we cannot definitively know these changes are a direct result of the endoparasitism, we can offer explanations for these changes based on knowledge of the behavior of endoparasites and the pathology associated with infection.
- 3. I think the limited age range of the dogs included in the study is important and should be mentioned early on in the manuscript - in the abstract, at least.
  - Thank you – we have added the age of the population in the abstract (line 25).
    - Line 23 – 28 now reads
    - This cross sectional study used complete blood count, serum biochemistry, and fecal flotation data collected from 3,018 **young dogs (<3 years of age**) enrolled within the Morris Animal Foundation Golden Retriever Lifetime Study (GRLS) to determine the prevalence of endoparasitism and compare bloodwork values of parasite positive and negative participants using logistic regression.
- 4. Likewise, the fact that the outcome and risk factor variables were assessed at the same appointment time is important and should be mentioned in the abstract and early in the paper. It could be argued that it's a cross-sectional study and not a retrospective study, as the study participants are not followed forward through time (as in a retrospective cohort study) or assessed "back through time" as in a retrospective case control study.
  - Thank you for your comment, I see what you are saying. Line 28 I have added:
    - Variables including age, gender, reproductive status, and geographic location at the time of initial evaluation were assessed to identify potential risk factors
  - We have also added a line to the beginning of the results to expressly state that all samples were collected at one time point.
    - Line 111-114: At a baseline visit, both blood and fecal samples were collected for a complete blood count (CBC), serum biochemistry, and fecal floatation analysis. From this data two models were created using either the results from the CBC (supplementary material 1) or serum biochemistry panel (supplementary material 2).
  - The authors agree that this is more of a cross sectional design and have altered the manuscript to reflect this. Major changes to the discussion are listed below with line numbers.
    - Line 193-195: Future studies are need to identify if the small changes identified within our data are due to parasite infection, or causative of the infection itself.
    - Line 199-201: As previously stated, additional prospective studies may help provide further evidence that the changes identified are attributable to endoparasitism.
- 4. The full logistic regression models were fit and then parameters were dropped based on AIC and likelihood ratio tests. Firstly, which of these two metrics did you use and/or how did you combine them? Also, how did you select these initial parameters for the model - since this is a very large study, I assume that other data was collected at the same time?
  - The authors agree this is important information and have added it to the methods section of the manuscript, Lines 299-304.
    - CBC and chemistry parameters were tested using the appropriate test (t-test, Mann-Whitney U test, Chi-Square test, or Fisher’s exact test) and selected for evaluation in the full model if *P*<0.5. Full models were fitted, then variables that did not achieve significance were removed. The final model was selected based on the lowest AIC score and likelihood ratio test indicating the least parameterize model was the best fit.
- 5. In the results you refer to the "chemistry model" and the "blood cell model" - I think since the results come before the methods it would be helpful to give more precise explanations.
  - Thank you for your comment, we agree and have added an explanation on the first line of each results paragraph as well as a line at the beginning of the results section.
    - Line 111-114: At a baseline visit, both blood and fecal samples were collected for a complete blood count (CBC), serum biochemistry, and fecal floatation analysis. From this data two models were created using either the results from the CBC (supplementary material 1) or serum biochemistry panel (supplementary material 2).
    - Line 138-140: A complete blood cell model was created to evaluate the association between parasite status and the following parameters: absolute eosinophils, absolute neutrophils, absolute lymphocytes, absolute monocytes, platelet count, RBC, and hemoglobin.
    - Line 150-152: A chemistry model was constructed to evaluate the association between the following parameters and parasite status: albumin, bilirubin, thyroxine (T4), glucose, sodium/potassium ratio, and total protein.
